# The value of HPV genotypes combined with clinical indicators in the classification of cervical squamous cell carcinoma and adenocarcinoma

**DOI:** 10.1186/s12885-022-09826-4

**Published:** 2022-07-15

**Authors:** Zhimin He, Rongsheng Chen, Shangying Hu, Yajiao Zhang, Yang Liu, Chengwei Li, Fajin Lv, Zhibo Xiao

**Affiliations:** 1 State Key Laboratory of Ultrasound in Medicine and Engineering, College of Biomedical Engineering, Chongqing Medical University, Chongqing, 400016 China; 2 Chongqing Key Laboratory of Biomedical Engineering, Chongqing Medical University, Chongqing, China; 3 Department of Radiology, The First Affiliated Hospital of Chongqing Medical University, Chongqing, 400016 PR China; 4 Department of Gynecology and Obstetrics, the University-Town Hospital of Chongqing Medical University, Chongqing, 401331 China; 5 College of Medical Informatics, Chongqing Medical University, Chongqing, 400016 China; 6 Institute of Medical Data, Chongqing Medical University, Chongqing, 400016 China

**Keywords:** Cervical cancer, Adenocarcinoma, Squamous cell carcinoma, Human papilloma virus, Tumour marker

## Abstract

**Background:**

To investigate the differences in HPV genotypes and clinical indicators between cervical squamous cell carcinoma and adenocarcinoma and to identify independent predictors for differentiating cervical squamous cell carcinoma and adenocarcinoma.

**Methods:**

A total of 319 patients with cervical cancer, including 238 patients with squamous cell carcinoma and 81 patients with adenocarcinoma, were retrospectively analysed. The clinical characteristics and laboratory indicators, including HPV genotypes, SCCAg, CA125, CA19-9, CYFRA 21–1 and parity, were analysed by univariate and multivariate analyses, and a classification model for cervical squamous cell carcinoma and adenocarcinoma was established. The model was validated in 96 patients with cervical cancer.

**Results:**

There were significant differences in SCCAg, CA125, CA19-9, CYFRA 21–1, HPV genotypes and clinical symptoms between cervical squamous cell carcinoma and adenocarcinoma (*P* < 0.05). Logistic regression analysis showed that SCCAg and HPV genotypes (high risk) were independent predictors for differentiating cervical squamous cell carcinoma from adenocarcinoma. The AUC value of the established classification model was 0.854 (95% CI: 0.804–0.904). The accuracy, sensitivity and specificity of the model were 0.846, 0.691 and 0.899, respectively. The classification accuracy was 0.823 when the model was verified.

**Conclusion:**

The histological type of cervical cancer patients with persistent infection of high-risk HPV subtypes and low serum SCCAg levels was more prone to being adenocarcinoma. When the above independent predictors occur, the occurrence and development of cervical adenocarcinoma should be anticipated, and early active intervention treatment should be used to improve the prognosis and survival of patients.

Cervical cancer is the most common malignant tumour of the female reproductive system, and it ranks fourth in both the number of new cases of cancer and the proportion of cancer deaths among women in the world. In 2020, there were approximately 604,000 new cases and 342,000 deaths worldwide, so cervical cancer poses a serious threat to the lives and health of women worldwide [1]. Squamous cell carcinoma (SCC) is the most common histological type of cervical cancer, accounting for 70% ~ 75% of cases, followed by adenocarcinoma (AC), accounting for 10% ~ 25% of cases [2]. A series of studies have shown that compared with concurrent squamous cell carcinoma, adenocarcinoma is more aggressive and less sensitive to radiotherapy and chemotherapy, with a higher metastasis rate, poorer prognosis and lower survival rate [3–9]. Therefore, many scholars believe that different clinical treatment strategies should be developed according to the different characteristics of cervical squamous cell carcinoma and adenocarcinoma, which is of great significance for determining accurate and personalized treatment plans [10–12]. Excitingly, new treatment strategies for cervical adenocarcinoma have been exploratory studies and applications. Noriyuki Okonogi et al. [13, 14] found that carbon-ion radiotherapy (CIRT) or concurrent cisplatin and CIRT showed promising results in the treatment of cervical adenocarcinoma, which may be a promising therapeutic strategy for cervical adenocarcinoma. The different epidemiology and prognosis of cervical squamous cell carcinoma and adenocarcinoma, as well as the individualized treatment options being explored, will inevitably lead to the need for new differential diagnosis methods for cervical squamous cell carcinoma and adenocarcinoma. Therefore, the differential diagnosis of cervical squamous cell carcinoma and adenocarcinoma is closely related to the treatment and prognosis of patients.

In 2021, the second edition of the *Screening and Treatment Guidelines for Cervical Precancerous Lesions* released by the World Health Organization (WHO) clearly recommended human papillomavirus (HPV) DNA testing as the preferred screening method for cervical cancer [15]. Persistent HPV infection is the main cause of cervical cancer, and there are many genotypes [16]. When HPV genotypes are 60%-70% nucleotide homologous, they cluster in the same species, and the most common HPV species, alpha 7 (HPV 18, 39, 45, 59, 68 and 70) and alpha 9 (HPV 16, 31, 33, 35, 52, 58 and 67), account for 80% of all cervical cancer cases [17]. HPV species are associated with the survival prognosis of cervical cancer patients. Cervical cancer patients who are HPV negative or only infected with HPV alpha 7 have a worse prognosis and higher risk; cervical cancer patients with coinfection of HPV alpha 7 and HPV alpha 9 are at medium risk; and cervical cancer patients infected with only HPV alpha 9 or other HPV genotypes are at lower risk [18]. Therefore, at present, risk subtypes based on the prognosis of HPV species are mostly used for the efficacy evaluation and prognosis prediction of radiotherapy and chemotherapy in patients with cervical cancer [18, 19], but their application in the differential diagnosis of cervical squamous cell carcinoma and adenocarcinoma has not been reported.

At present, clinical indicators such as tumour markers are often used in relevant studies on the identification of cervical squamous cell carcinoma and adenocarcinoma. Such indicators are easy to obtain clinically and play an important role in the differential diagnosis and prognosis prediction of tumours [20–25]. Lehtovirta P, Borras G, Liu Y et al. studied the differences in squamous cell carcinoma antigen (SCCAg) and carbohydrate antigen 125 (CA125), CA125 and carbohydrate antigen 19–9 (CA19-9), and SCCAg and CA19-9 levels between cervical squamous cell carcinoma and adenocarcinoma, and the results showed that the levels of CA125 and CA19-9 were higher in adenocarcinoma, while SCCAg had a higher level in squamous cell carcinoma [26–28]. However, the indicators used in these studies were not comprehensive, and there were intersections, but whether they are independent predictors of the differential diagnosis of cervical squamous cell carcinoma and adenocarcinoma has not been clarified. At the same time, it is not clear enough whether clinical indicators such as pregnancy, birth, body mass index (BMI), menopause, smoking history, clinical symptoms, and routine inflammatory indicators have any value in differentiating cervical squamous cell carcinoma from adenocarcinoma. Therefore, this study combined HPV genotypes with clinical indicators to provide a reference and basis for the noninvasive differential diagnosis of cervical squamous cell carcinoma and adenocarcinoma.

## Materials and methods

### Study patients

The inclusion criteria of this study were as follows: (1) the surgical method was radical hysterectomy and pelvic lymph node dissection, and cervical cancer was confirmed by postoperative pathology combined with immunohistochemistry; (2) preoperative chemotherapy, radiotherapy, coning or other treatments were not performed; and (3) the preoperative clinicopathological data were complete. The exclusion criteria were as follows: (1) other malignant tumours or major diseases; and (2) rare histological types of cervical cancer, such as adenosquamous carcinoma, clear cell carcinoma, or small cell carcinoma.

In this study, a total of 415 cases of cervical cancer admitted to the Department of Gynecology of the First Affiliated Hospital of Chongqing Medical University from January 2018 to September 2021 were enrolled according to the inclusion and exclusion criteria. The 319 cases (238 cases of squamous cell carcinoma and 81 cases of adenocarcinoma) from January 2018 to December 2020 were used as the primary cohort for retrospective analysis and establishing a clinical classification model; 96 cases (76 cases of squamous cell carcinoma and 20 cases of adenocarcinoma) from January 2021 to September 2021 were used as the validation cohort to verify the model effect. The demographics information of the study population are shown in Table 1. The study was approved by the Medical Ethics Committee of the First Affiliated Hospital of Chongqing Medical University (No.2021–395), the study being conducted according to the guidelines of the Declaration of Helsinki, and the informed consent of the subjects was exempted.

**Table 1:** The demographic information of the study population

Clinical Indicators	Primary cohort (*n* = 319)	Validation cohort (*n* = 96)	*P*
Age	49(43,56)	50(44,56)	0.286
BMI	22.89(21.08,24.75)	23.28(20.87,25.03)	0.638
Subtypes			0.361
SCC	238(74.6%)	76(79.2%)	
AC	81(25.4%)	20(20.8%)	
Menopausal Status			0.823
No	162(50.8%)	50(52.0%)	
Yes	157(49.2%)	46(48.0%)	
Clinical Symptoms			0.444
Asymptomatic or Other	69(21.7%)	25(26.0%)	
Contact Bleeding	166(52.0%)	43(44.8%)	
Irregular Vaginal Bleeding	84(26.3%)	28(29.2%)	
Smoking History			0.147
No	307(96.2%)	89(92.7%)	
Yes	12(3.8%)	7(7.3%)	
Gravidity	3(2,4)	3(2,5)	0.545
Parity	2(1,2)	2(1,2)	0.469
RBC	4.06(3.85,4.36)	4.15(3.92,4.38)	0.245
WBC	5.46(4.57,6.59)	5.05(4.35,6.09)	0.034^*^
PLT	209(174.00,242.00)	209(170.00,242.00)	0.566
Neutrophil Percentage	58.0(52.9,63.5)	54.9(50.9,60.1)	0.005^*^
Lymphocyte Percentage	31.1(26.3,36.3)	33.9(29.3,39.1)	0.004^*^
SCCAg	1.3(0.9,2.5)	2.0(1.0,2.9)	0.003^*^
CA125	14.6(11.0,22.8)	14.2(10.4,19.2)	0.369
CA19-9	10.9(7.4,17.2)	8.7(6.1,12.9)	0.005^*^
CYFRA 21–1	2.4(1.7,3.2)	2.4(1.9,3.4)	0.158
CEA	1.8(1.1,2.9)	1.9(1.3,3.2)	0.789
HPV Subtypes			0.253
Low Risk	233(73.1%)	78(81.2%)	
Medium Risk	16(5.0%)	4(4.2%)	
High Risk	70(21.9%)	14(14.6%)	

^*^A *p* value of < 0.05 was considered to indicate significant difference

### Clinical indicators and HPV genotypes

General clinical features and laboratory indicators included age, clinical symptoms, smoking history, parity, gravidity, menopausal status, BMI, red blood cell count (reference value range = 3.8–5.1, 10^12/L), white blood cell count (reference value range = 3.5–9.5, 10^9/L), platelets (reference value range = 101–320, 10^9/L), neutrophil percentage (reference value range = 40–75, %), lymphocyte percentage (reference value range = 20–50, %), SCCAg (reference value range = 0–2.7, ng/ml), CA125 (reference value range = 0–35, U/ml), CA19-9 (reference value range = 0–27, U/ml), CYFRA 21–1 (reference value range = 0–3.3, ng/ml), carcinoembryonic antigen (CEA, reference value range = 0.2–10, ng/ml), and HPV genotypes.

The above indicators were collected after the patients were admitted to hospital and before radical hysterectomy. Patients' blood routine data (RBC, WBC, platelets, neutrophil percentage, lymphocyte percentage) and tumour marker data (SCCAg, CA125, CA19-9, CYFRA 21–1, CEA) were obtained by blood samples drawn from veins. The patient's HPV-DNA test was carried out using cervical secretions and exfoliated cells of the cervix. The specific method was as follows: a disposable cervical sampler special cervical brush was placed in the cervical opening, rotated 5 times clockwise, put into 2 ml cell special preservation solution, fully rinsed, and then broken along the crease of the brush handle, leaving the brush head for examination.

The clinical symptoms were assessed as follows: contact bleeding, irregular vaginal bleeding, asymptomatic or other. For the HPV risk subtypes, high risk refers to HPV negative or only HPV alpha 7 positive; medium risk refers to both HPV alpha 7 and HPV alpha 9 being positive; and low risk refers to positive only for HPV alpha 9 or other HPV genotypes [18, 19].

### Statistical analysis

Statistical analysis was performed using SPSS statistical software version 22.0. The measurement data conforming to a normal distribution are presented as the mean ± standard deviation (SD), and the comparison between the two groups was performed by two independent sample *t* tests. If not normally distributed, the measurement data are expressed as the median (interquartile range), and significant differences between two groups were analysed using the Mann–Whitney U test. The qualitative data are represented as n (%) and were compared using the chi-square (χ^2^) test or Fisher's exact test. Spearman correlation analysis was used for correlation analysis. Independent predictors of cervical squamous carcinoma and adenocarcinoma were analysed by binary logistic regression. The Hosmer–Lemeshow test was used to analyse the goodness-of-fit of the model. Receiver operating characteristic (ROC) curves and calibration curves were drawn to evaluate the prediction efficiency of the model. *P* < 0.05 was considered statistically significant.

## Results

### Distribution of clinical indicators and HPV subtypes in squamous cell carcinoma and adenocarcinoma

**Fig. 1:**
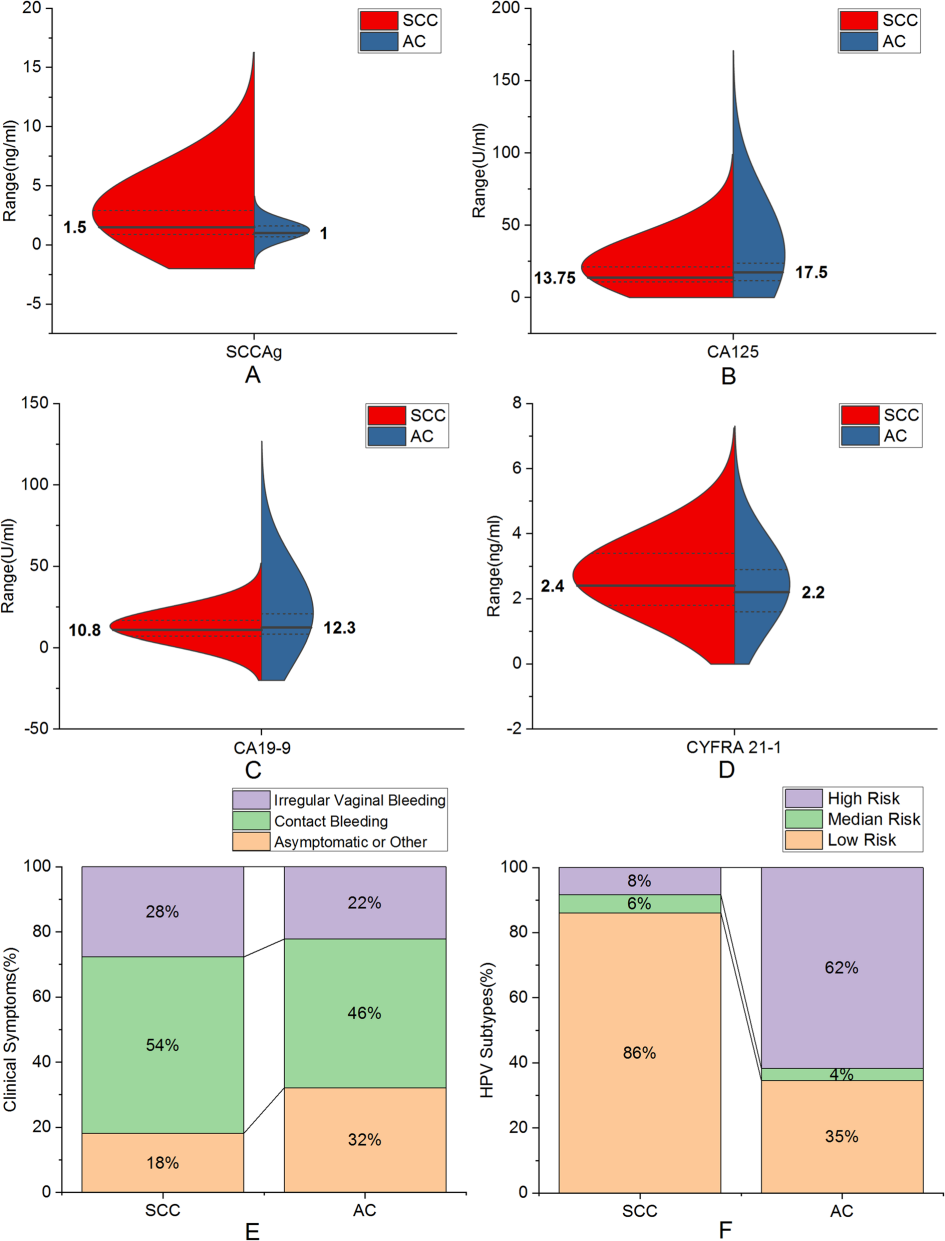
Split violin plot of the differences between cervical squamous cell carcinoma and adenocarcinoma in **A** SCCAg (Mann–Whitney U test; *P* < 0.001), **B** CA125 (Mann–Whitney U test; *P* = 0.031), **C** CA199 (Mann–Whitney U test; *P* = 0.030), **D** CYFRA 21–1 (Mann–Whitney U test; *P* = 0.023). The solid line represents the median, the dashed line represents the interquartile range, and the violin shape is the probability density estimated based on nuclear density in the split violin plot; Stacked histogram of the differences between cervical squamous cell carcinoma and adenocarcinoma in **E** clinical symptoms (chi-square test; *P* = 0.030), **F** HPV subtypes (chi-square test; *P* < 0.001)

The levels of CA125 and CA19-9 in the cervical adenocarcinoma group were higher than those in the squamous cell carcinoma group, while the level of CYFRA 21–1 was lower, and the differences were statistically significant (*P* < 0.05). The SCCAg level was significantly higher in cervical squamous cell carcinoma than in adenocarcinoma, and the difference was statistically significant (*P* < 0.001). The main clinical symptoms of the cervical squamous cell carcinoma group were contact bleeding (54.2%), followed by irregular vaginal bleeding (27.7%). The clinical symptoms of the adenocarcinoma group were mainly contact bleeding (45.7%), followed by asymptomatic or other symptoms (32.1%), and there was a significant difference between the two groups (*P* < 0.05). Regarding the HPV risk subtypes, the high-risk subtype (61.7%) was more common in the adenocarcinoma group, while the low-risk subtype (86.1%) was more common in the squamous cell carcinoma group, and there was a significant difference between the two groups (*P* < 0.05) (Fig. 1). HPV-negative patients accounted for 22.2% of patients with cervical adenocarcinoma and only 3.8% of patients with squamous cell carcinoma. Except for the above indicators, there was no significant difference in other indicators between the two groups (*P* > 0.05). The distribution of clinical indicators and HPV risk subtypes between squamous cell carcinoma and adenocarcinoma is shown in Table 2.

**Table 2:** Comparison of clinical indicators and HPV subtypes between SCC and AC

Clinical Indicators	SCC (*n* = 238)	AC (*n* = 81)	*P*
Age	50(43,56)	47(43,56)	0.509
Gravidity	3(2,4)	3(2,4)	0.813
Parity	1(1,2)	2(1,2)	0.092
BMI	22.89(21.08,24.93)	23.01(21.00,24.37)	0.456
RBC	4.08(3.88,4.38)	4.01(3.79,4.34)	0.205
WBC	5.47(4.56,6.58)	5.43(4.63,6.71)	0.706
PLT	209(173.00,241.57)	208(179.16,242.50)	0.959
SCCAg	1.5(0.9,2.9)	1(0.7,1.6)	< 0.001^*^
CA125	13.75(10.80,21.33)	17.50(11.55,24.85)	0.031^*^
CA19-9	10.8(7.08,16.80)	12.3(8.30,21.50)	0.030^*^
Neutrophil Percentage	58.1(53.00,63.50)	57.8(51.85,64.35)	0.913
Lymphocyte Percentage	31.45(27.10,36.43)	31(25.20,35.70)	0.349
CYFRA 21–1	2.4(1.78,3.40)	2.2(1.60,2.95)	0.023^*^
CEA	1.8(1.10,2.80)	1.9(1.30,3.85)	0.084
Menopausal Status			0.077
No	114(47.9%)	48(59.3%)	
Yes	124(52.1%)	33(40.7%)	
Smoking History			0.479
No	228(95.8%)	79(97.5%)	
Yes	10(4.2%)	2(2.5%)	
Clinical Symptoms			0.030^*^
Asymptomatic or Other	43(18.1%)	26(32.1%)	
Contact Bleeding	129(54.2%)	37(45.7%)	
Irregular Vaginal Bleeding	66(27.7%)	18(22.2%)	
HPV Subtypes			< 0.001^*^
Low Risk	205(86.1%)	28(34.6%)	
Medium Risk	13(5.5%)	3(3.7%)	
High Risk	20(8.4%)	50(61.7%)	

^*^A *p* value of < 0.05 was considered to indicate significant difference

### Independent Predictor Analysis of Squamous Cell Carcinoma and Adenocarcinoma

Binary logistic regression analysis was performed using the significantly different indicators between cervical squamous cell carcinoma and adenocarcinoma combined with clinical experience indicators as independent variables and the classification of the two as dependent variables. It was found that SCCAg (*P* = 0.009, OR = 0.671, 95% CI = 0.497–0.905) and HPV risk subtypes (*P* < 0.001, OR = 19.722, 95% CI = 9.252–42.040) were independent predictors for distinguishing cervical squamous cell carcinoma from adenocarcinoma. For each unit increase in the SCCAg level, the risk of the cervical cancer subtype being adenocarcinoma decreased by 0.671 times. Patients infected with high-risk HPV subtypes were 19.722 times more likely to develop adenocarcinoma of cervical cancer than those infected with low-risk HPV subtypes. The details are shown in Table 3.

**Table 3:** Independent predictor analysis of SCC and AC

Variable	B	S.E	Wald	*P*	*OR*	95% CI for OR
Parity	0.085	0.201	0.179	0.672	1.089	0.734–1.615
Menopausal Status	-0.426	0.369	1.327	0.249	0.653	0.317–1.348
Clinical Symptoms			2.804	0.246		
Contact Bleeding	-0.413	0.396	1.087	0.297	0.661	0.304–1.439
Irregular Vaginal Bleeding	-0.804	0.485	2.747	0.097	0.448	0.173–1.158
SCCAg	-0.399	0.153	6.814	0.009^*^	0.671	0.497–0.905
CA125	-0.001	0.005	0.031	0.861	0.999	0.989–1.010
CA19-9	0.030	0.016	3.539	0.060	1.030	0.999–1.063
CYFRA 21–1	-0.174	0.142	1.496	0.221	0.841	0.636–1.110
CEA	0.003	0.010	0.089	0.765	1.003	0.983–1.024
HPV			59.676	< 0.001^*^		
Medium Risk	0.895	0.724	1.526	0.217	2.445	0.592–10.119
High Risk	2.982	0.386	59.613	< 0.001^*^	19.722	9.252–42.040

^*^A *p* value of < 0.05 was considered to indicate significant difference

### Evaluation of model performance

**Fig. 2:**
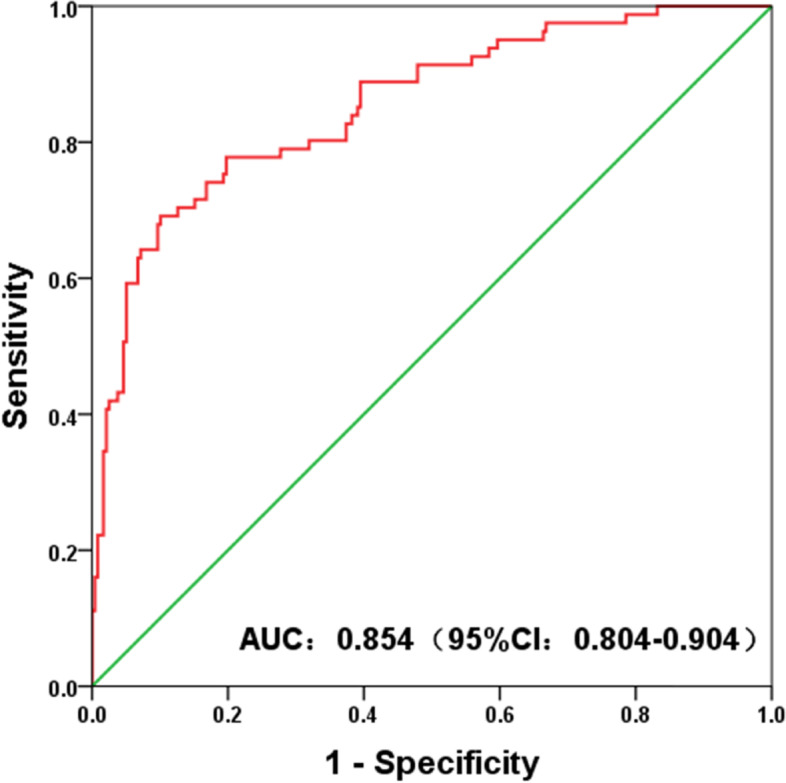
Receiver operating characteristic (ROC) curve analysis showed the effect of SCCAg combined with HPV subtypes on the classification of cervical squamous cell carcinoma and adenocarcinoma. The area under the curve (AUC) was 0.854 (95% CI: 0.804–0.904, *P* < 0.001)

**Fig. 3:**
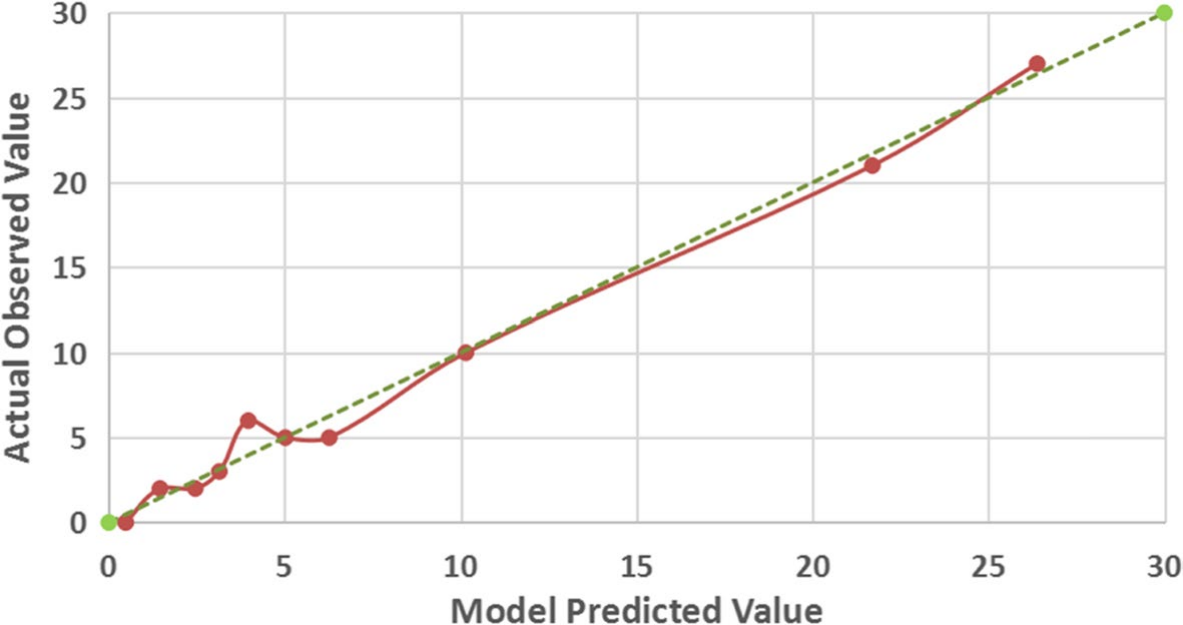
Calibration curve of the established model. It depicts the agreement between the model-predicted classification outcomes and the actual observed classification outcomes. The diagonal dotted line represents a perfect prediction by an ideal model. The red solid line represents the performance of the model, of which a closer fit to the diagonal dotted line represents a better prediction

According to the multivariate analysis of HPV risk subtypes and clinical indicators, a clinical classification model mainly based on SCCAg and HPV risk subtypes was established. The nonparametric Hosmer–Lemeshow test results (*P* = 0.963) indicated that the model had a high goodness of fit. A ROC curve was drawn for the classification model of cervical squamous cell carcinoma and adenocarcinoma. The area under the curve (AUC) was 0.854 (95% CI: 0.804–0.904, *P* < 0.001), and the model accuracy rate was 0.846. The prediction probability under the maximum Youden index (0.59) was taken as the cut-off value (cut-off = 0.277), and the sensitivity and specificity of the model were 0.691 and 0.899, respectively, as shown in Fig. 2. The calibration curve graph showed that the calibration curve was close to the ideal 45° curve, which indicated that the model had good calibration capabilities, as shown in Fig. 3. The data of cervical cancer patients from January 2021 to September 2021 were used for model validation, and the classification accuracy was 0.823, indicating that the model was stable and reproducible.

## Discussion

In current study, the age and distribution proportion of histological subtypes of the study population were consistent with previous studies and known knowledge [2]. In our study, the most common clinical symptom of cervical squamous cell carcinoma and adenocarcinoma was contact bleeding, while asymptomatic or other rare symptoms mostly occurred in adenocarcinoma, which was completely consistent with previous literature reports [8]. At the same time, the results showed that there were no significant differences between cervical squamous cell carcinoma and adenocarcinoma in age, gravidity, parity, BMI, menopause, smoking history, or routine inflammatory indicators (RBC, WBC, platelets, neutrophil percentage, and lymphocyte percentage) (*P* > 0.05). Among them, there was no significant difference in parity or BMI between cervical squamous cell carcinoma and adenocarcinoma, which was consistent with previous research results [29, 30]. However, previous studies suggested that smoking was significantly associated with the risk of cervical squamous cell carcinoma (RR = 1.50) but not with the risk of adenocarcinoma (RR = 0.86) [30]. We considered that the sample size of the smoking group in this study was too small to compare the difference in smoking history between cervical squamous cell carcinoma and adenocarcinoma. In addition, there was no significant difference between squamous cell carcinoma and adenocarcinoma in these clinical and conventional inflammatory indicators. We considered that cervical squamous cell carcinoma and adenocarcinoma are both malignant tumour subtypes of epithelial origin, and the application of conventional clinical and inflammatory indicators in the classification of tumour subtypes is limited and cannot be used for their differentiation [28, 31].

In the study of tumour markers, we jointly studied SCCAg, CA125, CA19-9, CYFRA 21–1 and CEA. The results showed that the levels of CA125 and CA19-9 in cervical adenocarcinoma were higher than those in squamous cell carcinoma, while the level of SCCAg in cervical squamous cell carcinoma was higher than that in adenocarcinoma; the difference was statistically significant (*P* < 0.05), which was consistent with previous research results [26–28]. There was no significant difference in CEA between them (*P* = 0.084), which was consistent with previous research results [27]. Meanwhile, the level of CYFRA 21–1 in cervical squamous cell carcinoma was higher than that in adenocarcinoma, and the difference was statistically significant (*P* < 0.05). This result may be because CYFRA 21–1 is a product of cytokeratin 19, which is mainly distributed in squamous and monolayer epithelial cells [32]. Multivariate analysis further confirmed that SCCAg was an independent predictor of cervical squamous cell carcinoma and adenocarcinoma (*P* = 0.009, OR = 0.671, 95% CI = 0.497–0.905). SCCAg is a subcomponent of TA-4 extracted from cervical squamous cell carcinoma, and its serum level can be used as one of the auxiliary indicators for the diagnosis, efficacy evaluation and prognosis prediction of cervical squamous cell carcinoma [23–25, 33–35]. Therefore, we believe that serum SCCAg levels can play a good role in the differentiation of cervical squamous cell carcinoma and adenocarcinoma.

In the correlation study of HPV risk subtypes and the identification of cervical squamous cell carcinoma and adenocarcinoma, the results of this study showed that the HPV-negative rate of cervical adenocarcinoma was approximately 22.2%, while that of cervical squamous cell carcinoma was approximately 3.8%. This is consistent with the results in previous studies that approximately 20%-30% of patients with cervical adenocarcinoma were HPV negative, while only approximately 5% of patients with cervical squamous cell carcinoma were HPV negative [36–38]. Moreover, cervical cancer patients persistently infected with high-risk HPV subtypes tended to have adenocarcinoma, while cervical cancer patients persistently infected with low-risk HPV subtypes tended to have squamous cell carcinoma (*P* < 0.001). That is, cervical adenocarcinoma is more likely to show no HPV infection or only HPV alpha 7 (HPV 18, 39, 45, 59, 68 and 70) positivity, whereas cervical squamous cell carcinoma is more likely to show only HPV alpha 9 (HPV 16, 31, 33, 35, 52, 58 and 67) positivity or positivity of other HPV genotypes. This result may be because HPV 18, as the most common genotype of HPV alpha 7, is most associated with cervical adenocarcinoma, while HPV 16, as the most common genotype of HPV alpha 9, is closely related to cervical squamous cell carcinoma [39, 40]. In further multivariate analysis, we found that the HPV risk subtype was also an independent predictor for differentiating cervical squamous cell carcinoma from adenocarcinoma. Compared with patients infected with low-risk HPV subtypes, patients infected with high-risk HPV subtypes were approximately 19 times more likely to develop adenocarcinoma (*P* < 0.001, OR = 19.722, 95% CI = 9.252–42.040). Patients with high-risk cervical cancer who are HPV negative or only infected with HPV alpha 7 have a worse clinical prognosis [18, 19, 41]. Our results showed that the histological type of these cervical cancer patients was more prone to being adenocarcinoma. This is consistent with the clinical characteristics of adenocarcinoma, with more aggressiveness, insensitivity to radiotherapy and chemotherapy, a higher metastasis rate, poorer prognosis and a lower survival rate compared with the same period of squamous cell carcinoma [3–9]. Therefore, we believe that HPV risk subtypes have good classification ability between cervical squamous cell carcinoma and adenocarcinoma.

At the same time, this study established a clinical classification model for the differential diagnosis of cervical squamous cell carcinoma and adenocarcinoma based on SCCAg and HPV risk subtypes, and the ROC curve was drawn. The AUC of the model was 0.854 (95% CI: 0.804–0.904, *P* < 0.001), and the model accuracy was 0.846. The prediction probability under the maximum Youden index was taken as the cut-off value, and the sensitivity and specificity of the model were 0.691 and 0.899, respectively. Nonparametric Hosmer–Lemeshow test results (*P* = 0.963) indicated that the model had a high goodness of fit, suggesting that the classification model had good differentiation and calibration abilities and could distinguish cervical squamous cell carcinoma and adenocarcinoma well. In addition, the data of cervical cancer patients from January 2021 to September 2021 were used for model validation, and the classification accuracy was 0.823, indicating the good stability and reproducibility of the model.

Of course, this study also has some limitations. (1) As a retrospective study, this study has a certain selection bias. For example, the sample size of the smoking group was small, which makes it difficult to evaluate the difference in smoking history between cervical squamous cell carcinoma and adenocarcinoma. (2) Imaging features were not included in this study, and we only explored the differences in clinical features and laboratory parameters between cervical squamous cell carcinoma and adenocarcinoma. In the future, we will combine imaging features for further research.

In conclusion, HPV risk subtypes and SCCAg are independent predictors for differentiating cervical squamous cell carcinoma from adenocarcinoma and can play a good role in classification. The histological type of cervical cancer patients with persistent infection of high-risk HPV subtypes and low serum SCCAg levels is more prone to being adenocarcinoma, while the histological type of cervical cancer patients with persistent infection of low-risk HPV subtypes and high serum SCCAg levels tends to be squamous cell carcinoma. Clinical attention should be given to the occurrence and development of cervical adenocarcinoma, and early intervention should be given to improve the prognosis and survival of patients.

## Data Availability

The datasets used and/or analyzed during the current study available from the corresponding author on reasonable request.

## Abbreviations

AC: Adenocarcinoma
SCC: Squamous cell carcinoma
HPV: Human papillomavirus
SCCAg: Squamous cell carcinoma antigen
CA125: Carbohydrate antigen 125
CA19-9: Carbohydrate antigen 19–9
BMI: Body mass index
CEA: Carcinoembryonic antigen
RBC: Red blood cell count
WBC: White blood cell count
PLT: Platelets
OR: Odds ratio
ROC: Receiver operating characteristic
AUC: The area under the curve
